# Supplementary material for: KRAS drives immune evasion in a genetic model of pancreatic cancer
Source: Nat Commun. 2021 Mar 5;12:1482. doi: 10.1038/s41467-021-21736-w (PMC7935870; doi:10.1038/s41467-021-21736-w)
Supplement: Supplementary file 1 — Supplementary Information [file 41467_2021_21736_MOESM1_ESM.pdf]

Supplementary Table 1

Genes with significantly greater expression in the epithelial cells of KRAS intact compared to KRAS KO tumors. Statistical significance was determined using the non-parametric Wilcoxon rank sum test, and the p-value was adjusted for multiple comparisons using the Bonferroni correction.

Gene	*p* value
ANXA10	6.51338E-93
APOC1	4.53496E-20
ATP1B1	6.50718E-61
CCNB2	5.85437E-24
CELA2A	3.92155E-23
CELA3B	5.56608E-23
CENPW	2.5981E-34
CFI	2.84673E-79
CLDN2	1.065E-111
CLDN6	3.3676E-143
CSRP1	4.95644E-23
ELF3	1.0607E-177
FOXQ1	8.92881E-49
FSTL1	1.41524E-18
FXYD3	7.9286E-153
GADD45G	3.51533E-75
GAP43	2.43799E-91
GCHFR	1.57054E-36
GMNN	2.08914E-57
GPX2	4.3354E-112
HIST1H1C	1.1261E-55
HS3ST1	1.67235E-41
ICAM1	9.6954E-175
KIF23	3.92944E-65
KRT18	4.62388E-28
LAMA4	7.07337E-32
MGST2	9.86953E-53
MUC1	5.31276E-64
SEC61B	2.18567E-19
SPINT2	1.96566E-23
STMN1	4.65657E-42
TINAGL1	2.21136E-73
TM4SF4	7.83744E-30
TSPAN13	1.68071E-66
TUBA4A	3.67516E-22
TUBB3	7.93651E-23

Supplementary Table 2

List of antibodies and primers

Antibodies	Source	Identifier
Ras (G12D Mutant Specific) (D8H7) Rabbit mAb (dilution 1:2000)	Cell Signaling Technology	Cat#14429
Rabbit polyclonal anti-AKT (dilution 1:2000)	Cell Signaling Technology	Cat#9272
Phospho-Akt (Thr308) (244F9) Rabbit mAb (dilution 1:500)	Cell Signaling Technology	Cat#4056
Phospho-Akt (Ser473) (D9E) XP® Rabbit mAb (dilution 1:500)	Cell Signaling Technology	Cat#4060
B-Raf (55C6) Rabbit mAb (dilution 1:500)	Cell Signaling Technology	Cat#9433
c-Raf Rabbit Antibody (dilution 1:500)	Cell Signaling Technology	Cat#9422
Phospho-MEK1/2 (Ser217/221) Rabbit Antibody (dilution 1:1000)	Cell Signaling Technology	Cat#9121
p44/42 MAPK (Erk1/2) Rabbit Antibody (dilution 1:1000)	Cell Signaling Technology	Cat#9102
Phospho-p44/42 MAPK (Erk1/2) (Thr202/Tyr204) (D13.14.4E) XP® Rabbit mAb (dilution 1:1000)	Cell Signaling Technology	Cat#4370
PI3 Kinase p110α (C73F8) Rabbit mAb (dilution 1:1000)	Cell Signaling Technology	Cat#4249
Phospho-GSK-3α/β (Ser21/9) Antibody (dilution 1:500)	Cell Signaling Technology	Cat#9331
Keratin 17/19 (D32D9) Rabbit mAb #3984 (dilution 1:1000)	Cell Signaling Technology	Cat#3984
Sox9 (D8G8H) Rabbit mAb #82630 (dilution 1:1000)	Cell Signaling Technology	Cat#82630
K-Ras (F234) mAb (dilution 1:200)	Santa Cruz Biotechnology	Cat#sc-30
c-Myc Rabbbit Antibody (N-262) (dilution 1:1000)	Santa Cruz Biotechnology	Cat#sc-764
Anti-phospho-c-Myc (Thr58/Ser62) Rabbit mAb (dilution 1:2000)	Millipore Sigma	Cat#04-217
Anti-MAP Kinase 2/Erk2 Antibody clone 1B3B9 mAb (dilution 1:2000)	EMD Millipore	Cat#05-157
Anti-PDX-1 mouse mAb (dilution 1:2000)	BD Biosciences	Cat#562160
Anti-ERK1/2 Phospho (Thr202/Tyr204) Antibody (dilution 1:200)	BioLegend	Cat#675502
CD326 (EpCAM) Monoclonal Antibody (1B7), PE (dilution 1:1000)	eBioscience	Cat#12-9326-42
Ly-6A/E (Sca-1) Monoclonal Antibody (D7), APC (dilution 1:1000)	eBioscience	Cat#17-5981-81
Anti-PD1 (dilution 1:10)	BioXCell	Cat#BE0146
Anti-CTLA4 (dilution 1:10)	BioXCell	Cat#BE0164
Oligonucleotides		
5-CAGTTCTCTGCAGTTGTTGG-3	this study	KRAS genotyping 1
5-TCCGAATTCAGTGACTACAGATGTACAGAG-3	this study	KRAS genotyping 2
5-TCCAACACAGATGTTCTTAGGCTAC-3	this study	KRAS genotyping 3
5-GGGTAGGTGTTGGGATAGCTG-3	this study	KRAS sequencing 1
5-GCAGACTGTAGAGCAGCGTTAC-3	this study	KRAS sequencing 4
5-GGAACTAAAGGACATCAC-3	this study	KRAS sequencing 7

a

LSL $KRAS^{G12D}$ p53KO mice
↓
Primary organotypic culture
↓
Retroviral self-excising Cre
FACS sorting EpCAM+
↓
$KRAS^{G12D}$ p53KO epithelial cells
↓
Clonal $KRAS^{G12D}$ p53KO cell lines

Clonal $KRAS^{G12D}$ cell lines
↓
Orthotopic or subcutaneous tumors
↓
Tumor and metastases-derived cell lines
↓
$KRAS^{G12D}$ gRNA
CRISPR/Cas9
↓
Clonal KRAS ablated cell lines

b

ID	Derivation	Genotype	Origin
KC1 (P2)	pre-tumor	$KRAS^{G12D/+}$ p53KO	Ischenko et al., 2014[1]
KC2 (P2/tu1)	tumor	$KRAS^{G12D/+}$ p53KO	Ischenko et al., 2014[1]
KC3 (P2/met1)	metastasis	$KRAS^{G12D/+}$ p53KO	Ischenko et al., 2014[1]
KC4 (P16)	pre-tumor	$KRAS^{G12D/-}$ p53KO	Ischenko et al., 2014[1]
KC5 (P16/tu1)	tumor	$KRAS^{G12D/-}$ p53KO	Ischenko et al., 2014[1]
KC6 (P16/met1)	metastasis	$KRAS^{G12D/-}$ p53KO	Ischenko et al., 2014[1]
KC7 (P26)	pre-tumor	$KRAS^{G12D/+}$ p53KO	Ischenko et al., 2014[1]
KC8 (P26/tu4)	tumor	$KRAS^{G12D/+}$ p53KO	Ischenko et al., 2014[1]
KC9 (P26/met1)	metastasis	$KRAS^{G12D/+}$ p53KO	Ischenko et al., 2014[1]
KC10 (P28)	pre-tumor	$KRAS^{G12D/+}$ p53KO	Ischenko et al., 2014[1]
KC11 (P28/tu1)	pre-tumor	$KRAS^{G12D/+}$ p53KO	Ischenko et al., 2014[1]
KPC (FC1245)	tumor	$KRAS^{G12D/+}$ $p53^{R172H/+}$	Hingorani et al., 2005[2]
KPC (FC1199)	tumor	$KRAS^{G12D/+}$ $p53^{R172H/+}$	Hingorani et al., 2005[2]
KPC (FC1242)	tumor	$KRAS^{G12D/+}$ $p53^{R172H/+}$	Hingorani et al., 2005[2]
KPC (KPC1)	tumor	$KRAS^{G12D/+}$ $p53^{R172H/+}$	Sivaram et al., 2019[3]
iKRAS1 (A9312)	tumor	TetO-$KRAS^{G12D/+}$ $p53^{R172H/+}$	Collins et al., 2012[4]
iKRAS2 (A9993)	tumor	TetO-$KRAS^{G12D/+}$ $p53^{R172H/+}$	Collins et al., 2012[4]

Supplementary Figure 1

c

Indels induced by CRISPR/Cas9 sgRNA

```
sgKRAS                                           GTGGTTGGAGCTGATGGCGT
KRAS G12D  ATGACTGAGTATAAGCTTGTGGTGGTTGGAGCTGATGGCGTAGGC
KRAS WT    ATGACTGAGTATAAACTTGTGGTGGTTGGAGCTGGTGGCGTAGGC
CL2        ATGACTGAGTATAAGCTTGTGGTGGTTGGAGCTGATGG-GTAGGC
CL3        ATGACTGAGTATAAGCTTGTGGTGGTTGGAGCTGAT--CGTAGGC
CL4        ATGACTGAGTATAAGCTTGTGGTGGTTG------(19bp)-----
CL5        ATGACTGAGTATAAGCTTGTGGTGGTTGGAGCTGAT-GCGTAGGC
CL6        ATGACTGAGTATAAGCTTGTGGTGGTTGGAGCTG--GGCGTAGGC
CL7        ATGACTGAGTATAAGCTTGTGGTGGTTGGAGCTGATG-(19bp)-
           M  T  E  Y  K  L  V  V  V  G  A  D  G  V  G
```

d

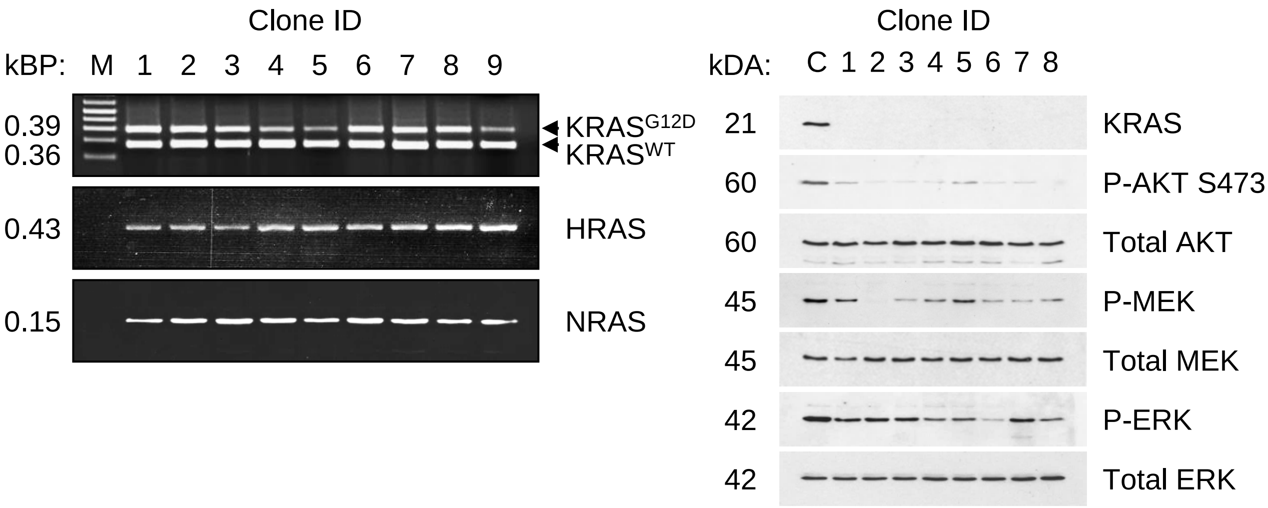

e

Subcutaneous, nude mice				
No	Cell type	KRAS status	Clone ID	Number of mice
1	KC	intact	P2	6
2	KC	intact	P16	6
3	KC	intact	P18	6
4	KC	intact	P26	6
5	KC	intact	P28	6
6	KC	KO	P2/5	2
7	KC	KO	P16/met4	2
8	KC	KO	P16/met6	2
10	KC	KO	P26/tu4	2
11	KPC	intact	FC1245	4
12	KPC	intact	FC1199	1
13	KPC	intact	FC1242	1
14	KPC	intact	KPC1	1
15	KPC	KO	FC1245/B4	2
16	KPC	KO	FC1245/B4/1	2
17	KPC	KO	FC1245/B4/5	1
18	KPC	KO	FC1245/B10	1
19	KPC	KO	FC1245/B17	1
20	KPC	KO	FC1199/11/7	3

Orthotopic, nude mice				
No	Cell type	KRAS status	Clone ID	Number of mice
1	KC	intact	P2	3
2	KC	intact	P16	3
3	KC	intact	P26/1	2
4	KC	Intact	P16/tu4	2
5	KC	KO	P26/1/3	2
6	KC	KO	P26/1/4	2
7	KC	KO	P26/1/7	1
8	KPC	intact	FC1245	4
9	KPC	KO	FC1245/B4/1	5
10	KPC	KO	FC1245/B4/5	1
11	KPC	KO	FC1199/11/7	1

Orthotopic, wild-type mice				
No	Cell type	KRAS status	Clone ID	Number of mice
1	KPC	intact	FC1245	16
2	KPC	intact	FC1245/B2	4
3	KPC	intact	FC1245/B22	1
4	KPC	intact	FC1245/B25	1
5	KPC	intact	FC1199	4
6	KPC	intact	FC1199/11/6	2
7	KPC	intact	FC1242	4
8	KPC	intact	KPC1	7
9	KPC	KO	FC1245/B4	11
10	KPC	KO	FC1245/B4/1	30
11	KPC	KO	FC1245/B4/5	10
12	KPC	KO	FC1245/B10	1
13	KPC	KO	FC1245/B17	2
14	KPC	KO	FC1199/11/7	2
15	KPC	KO	FC1199/11/12	1

Supplementary Figure 1

Supplementary Figure 1.

a. Generation of pancreatic epithelial cells from LSL KRASG12D p53-/- mice, referred to as LSL KRASG12D p53KO, which carry a latent point mutant of the KRAS2 gene. To delete the transcriptional termination cassette (Lox-Stop-Lox) and thus activate expression of the KRASG12D protein, we used a self-excising retroviral vector expressing Cre recombinase. The cells were passaged over a period of ≥ 2 months and monitored for acquisition of a malignant phenotype defined as the potential to generate subcutaneous tumors in nude mice. Cancer cell lines were derived from the resected tumors.

b. Genotypes of precancerous and tumor-derived KRASG12D cell lines used in this study.

c. Sequence analysis showing deletions induced by CRISPR in KRAS KO clones.

d. Genomic pcr (left) showing unaltered NRAS and HRAS loci in KRAS KO clones and Western blot analysis (right) showing stable loss of KRAS expression.

e. The experimental generation of KRAS intact and deficient tumors in mice.

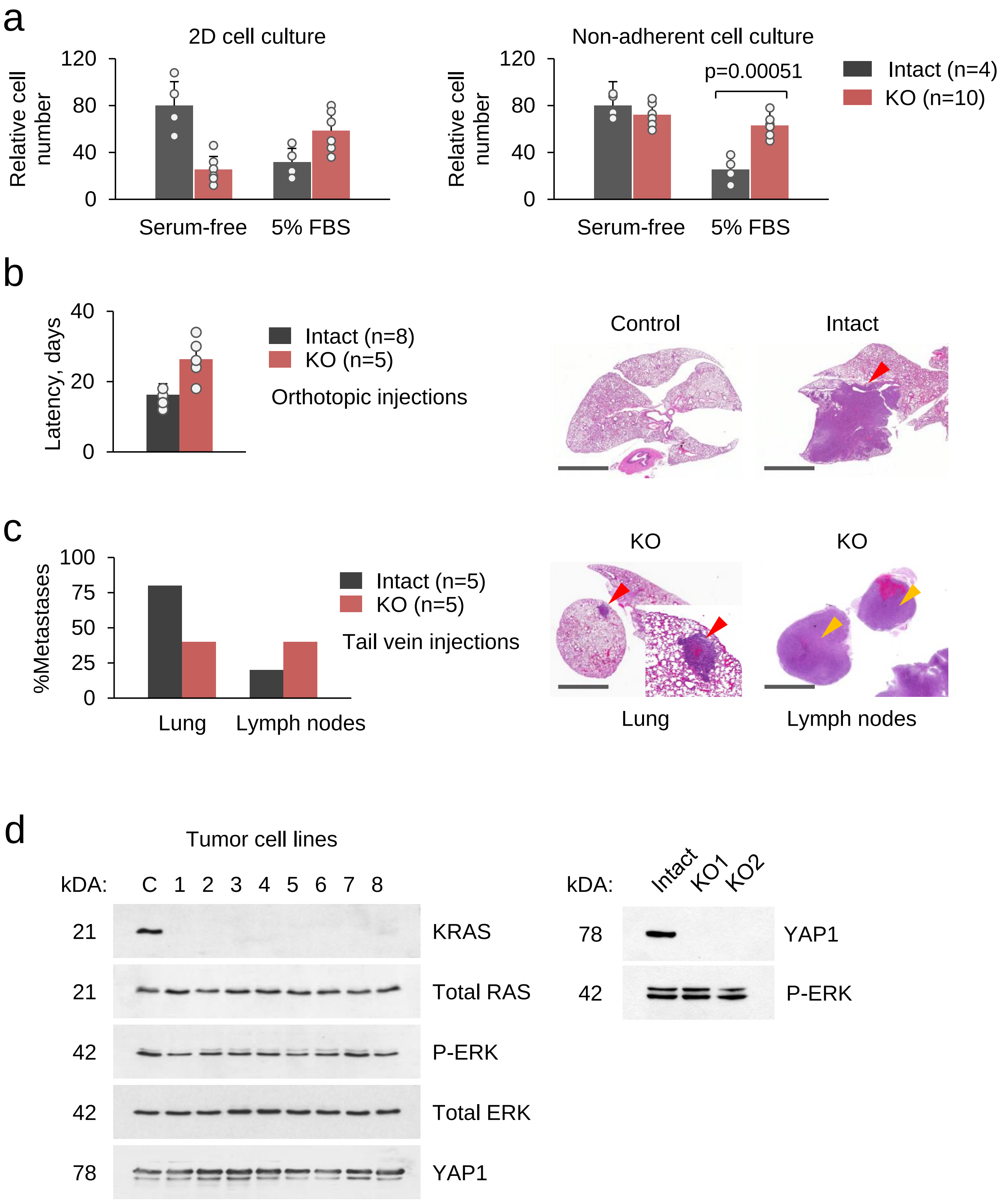

Supplementary Figure 2

e

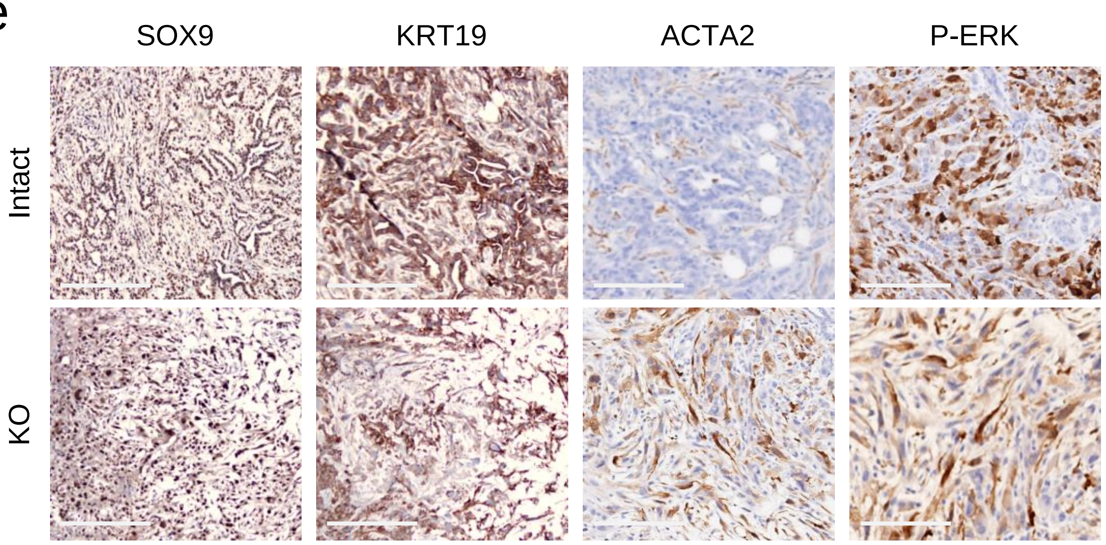
SOX9
KRT19
ACTA2
P-ERK
Intact
KO

f

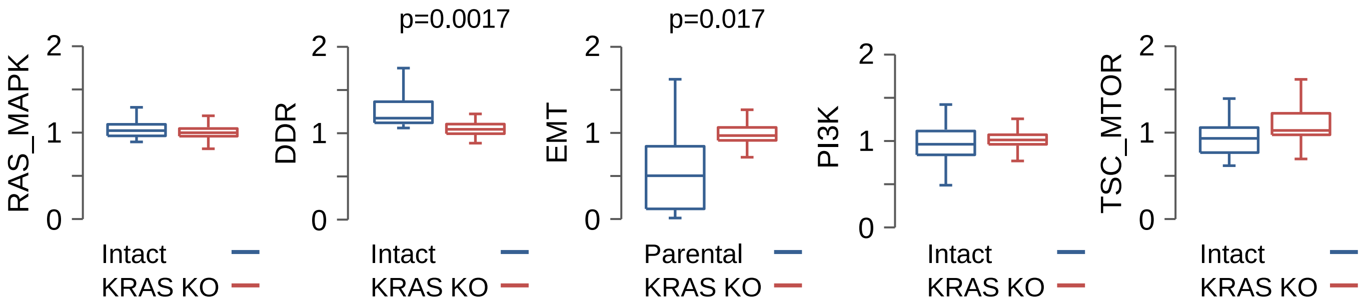
p=0.0017
p=0.017
RAS_MAPK
DDR
EMT
PI3K
TSC_MTOR
0
1
2
Intact
KRAS KO
Parental

g

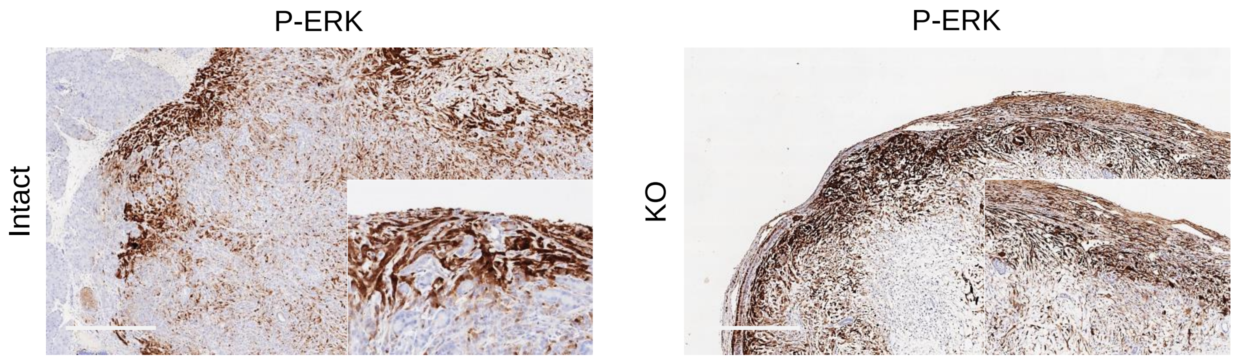
P-ERK
P-ERK
Intact
KO

h

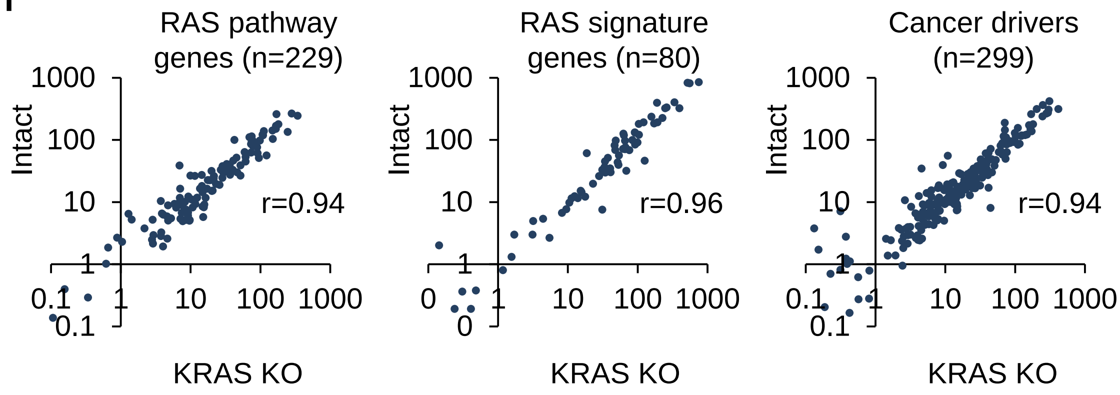

Supplementary Figure 2.

a. Growth (left) and viability (right) of KRAS intact (n=4) and KRAS KO KC cells (n=10) were assessed in serum-free CnT medium and DME containing 5% FBS. Cumulative cell numbers after 2 weeks in culture are shown. Error bars represent the standard deviation. Significance was determined using two-tailed test at the 0.05 confidence interval.

b. Latency of orthotopic tumors in nude mice formed by KRAS intact and KRAS KO KC cells.

c. Lung and lymph node metastases formed by KRAS intact and KRAS KO KC cells following tail vein injections into nude mice (arrowheads). Control non-tumorigenic lung is shown. Bar graph displays the quantification of mice with metastatic foci. Scale bar 3mm.

d. Western blot analysis of tumor-derived KRAS KO cell lines shows stable loss of KRASG12D expression. CRISPR-mediated knockout of YAP1 showed no effect on ERK activation (right).

e. Immunohistochemistry of KRAS intact and KRAS KO tumors in nude mice. Scale bar 200 μM.

f. Box plots of pathway activity scores as assessed by RPPA of KRAS intact and KRAS KO KC cell lines (n=6). The box plots show center line as median, box limits as upper and lower quartiles, and whiskers represent 1.5 × interquartile range (IQR). Error bars represent the standard deviation. Significance was determined using two-tailed test at the 0.05 confidence interval.

g. Immunohistochemistry staining for P-ERK using KRAS intact and KRAS KO KC tumors. Scale bar 600 μM.

h. Scatter plots comparing gene signature scores of RAS pathway genes, KRAS signature genes and TCGA cancer driver genes in KRAS intact and KRAS KO KPC cells with Pearson correlation coefficients.

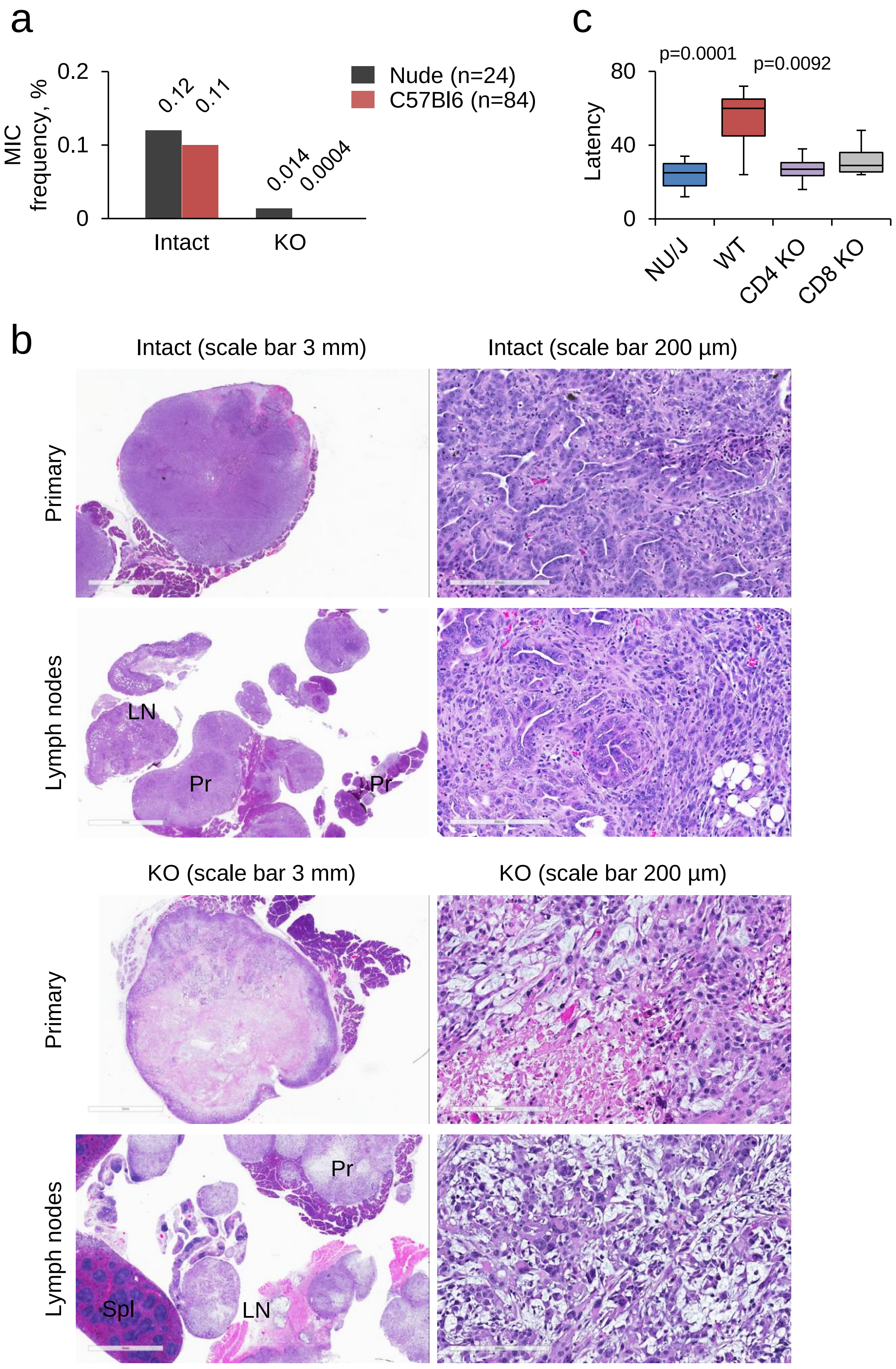

Pr, primary tumor; Spl. Spleen; LN, lymph node

Supplementary Figure 3

d

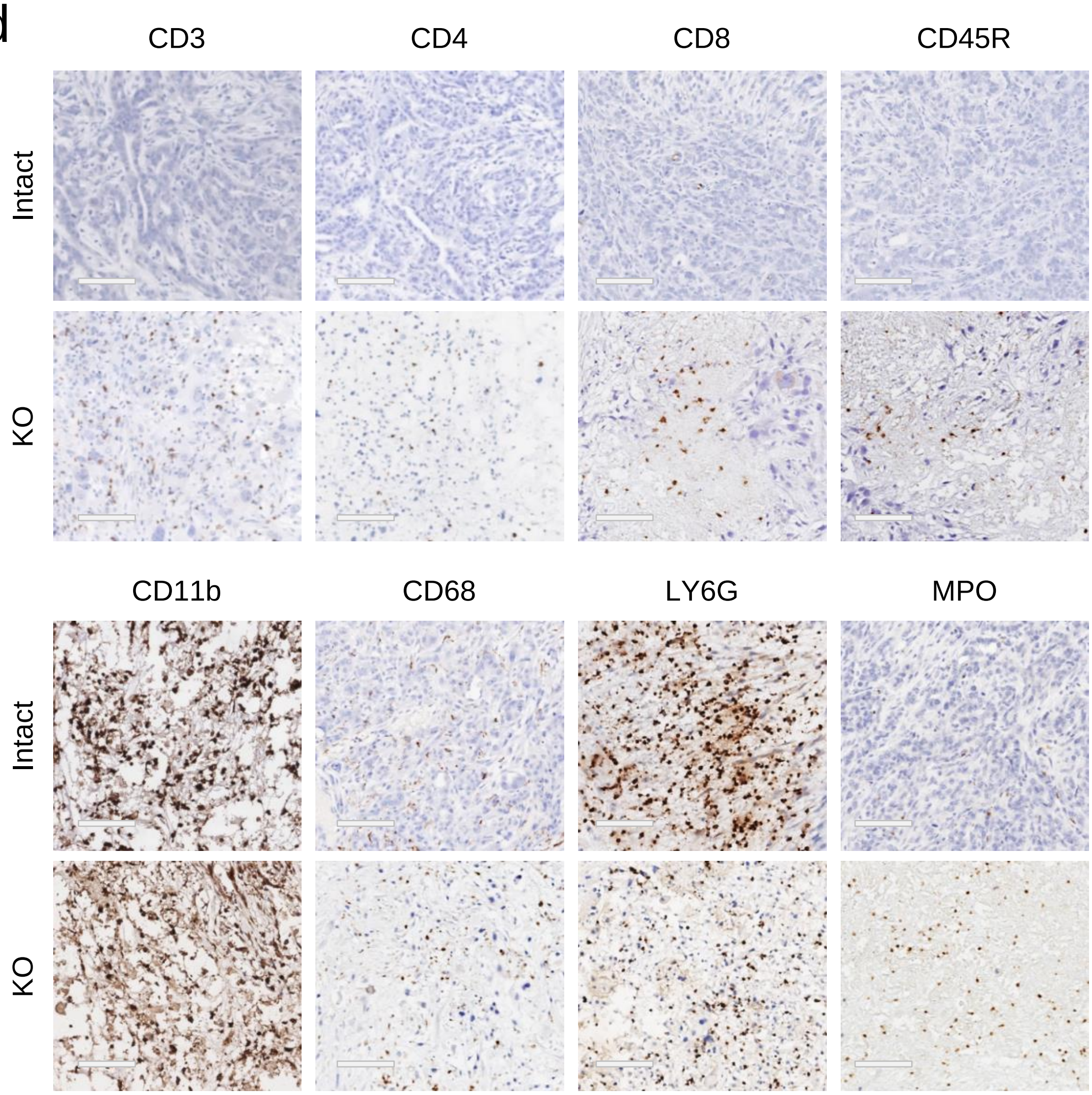

e

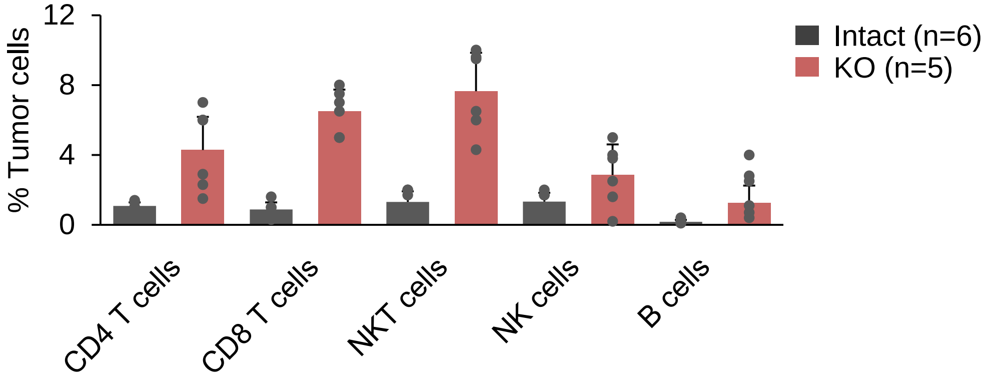
12
8
4
0
% Tumor cells
Intact (n=6)
KO (n=5)
CD4 T cells
CD8 T cells
NKT cells
NK cells
B cells

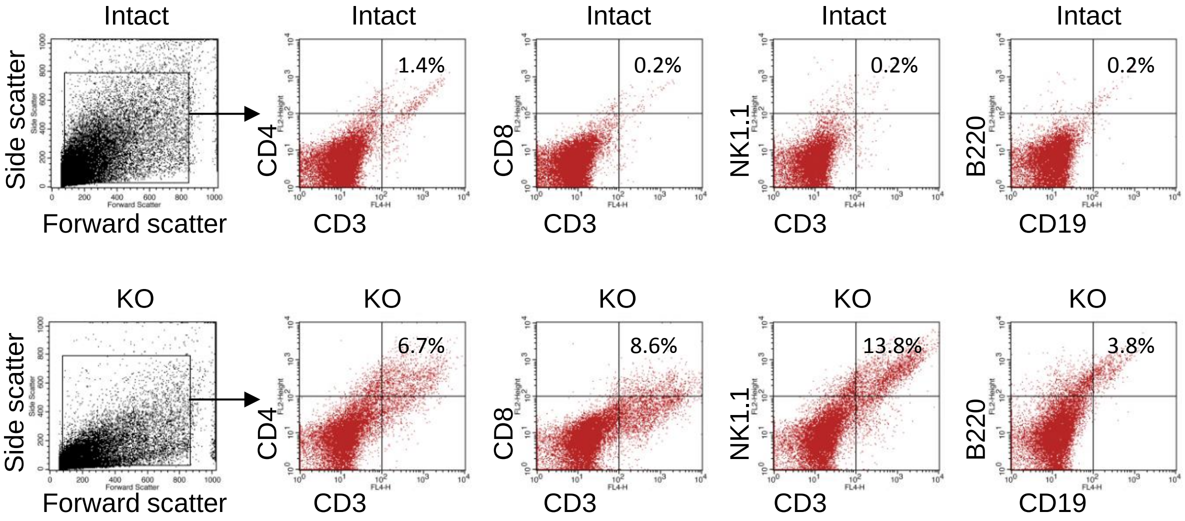
Intact
Side scatter
Forward scatter
Intact
1.4%
CD4
CD3
Intact
0.2%
CD8
CD3
Intact
0.2%
NK1.1
CD3
Intact
0.2%
B220
CD19
KO
Side scatter
Forward scatter
KO
6.7%
CD4
CD3
KO
8.6%
CD8
CD3
KO
13.8%
NK1.1
CD3
KO
3.8%
B220
CD19

Supplementary Figure 3

a. Quantification of metastasis-initiating cell frequency (MIC) in KRAS intact and KRAS KO KPC cells in nude and wild-type mice.

b. Hematoxylin and eosin staining of primary tumors and lymph node metastases formed by KRAS intact and KRAS KO KPC cells following orthotopic injections into wild-type mice.

c. Comparison of KRAS KO tumor development in NU/J, WT, CD4 KO and CD8 KO mice (n=4 for each genotype). Box plots show center line as median, box limits as upper and lower quartiles, and whiskers represent 1.5 × interquartile range (IQR). Significance was determined using two-tailed t-test at the 0.05 confidence interval.

d. Immunohistochemistry of tumor-infiltrating lymphocytes in KRAS intact and KRAS KO tumors. Scale bar 100 μM.

e. Flow cytometric analysis of tumor-infiltrating lymphocytes in KRAS intact and KRAS KO tumors. Lymphocyte subsets were defied as CD4 T cells (CD3+ CD4+), CD8 T cells (CD3+ CD8+), NKT cells (CD3+ NK1.1+), NK cells (CD3− NK1.1+), and B cells (CD19+ B220+). The gating strategy is shown (bottom panels). Data are expressed as mean ± SD.

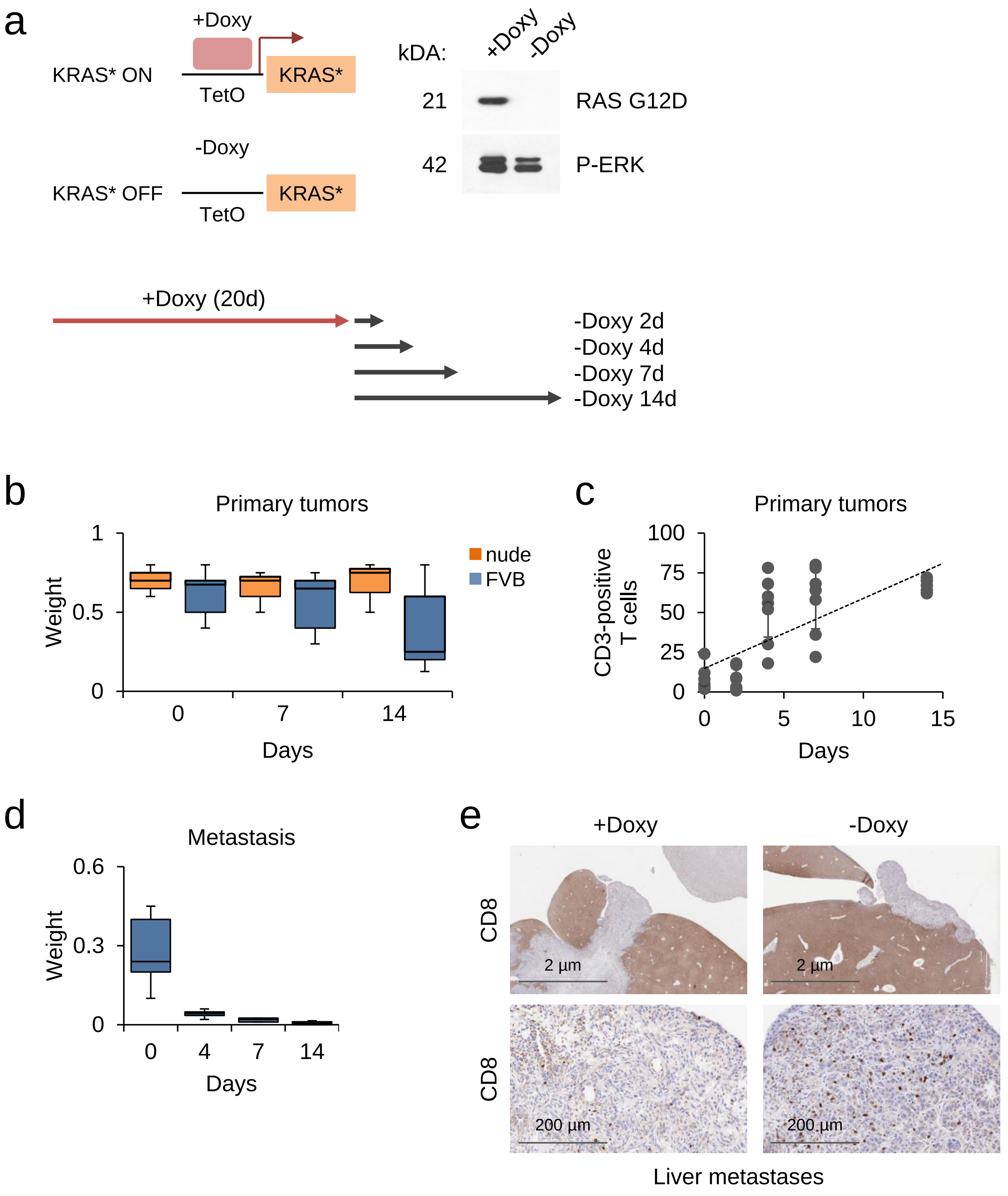
a
+Doxy
KRAS* ON
TetO
KRAS*
-Doxy
KRAS* OFF
TetO
KRAS*
kDA:
+Doxy
-Doxy
21
RAS G12D
42
P-ERK
+Doxy (20d)
-Doxy 2d
-Doxy 4d
-Doxy 7d
-Doxy 14d
b
Primary tumors
Weight
1
0.5
0
0
7
14
Days
nude
FVB
c
Primary tumors
CD3-positive T cells
100
75
50
25
0
0
5
10
15
Days
d
Metastasis
Weight
0.6
0.3
0
0
4
7
14
Days
e
+Doxy
-Doxy
CD8
CD8
2 µm
2 µm
200 µm
200 µm
Liver metastases

f

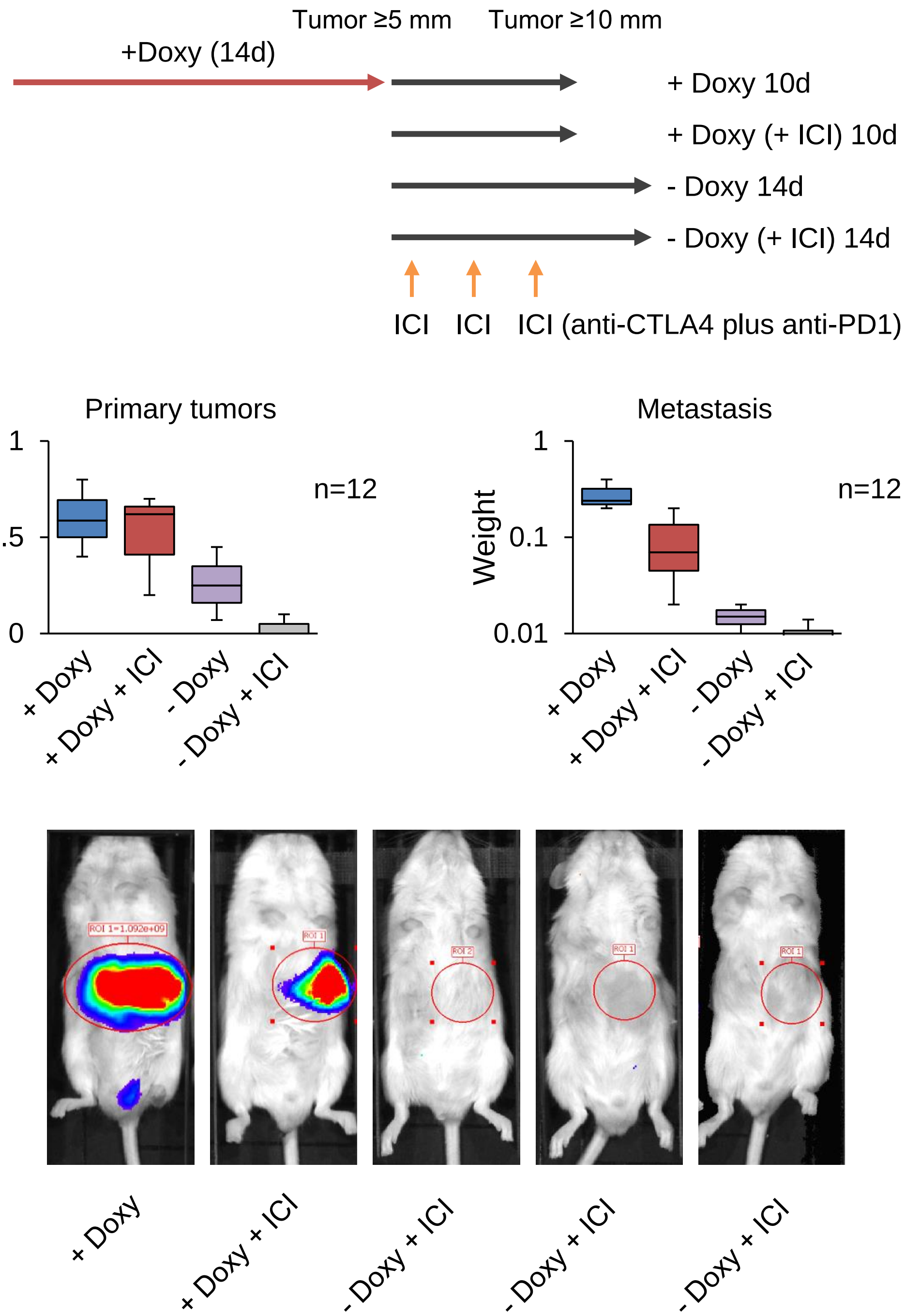
Tumor ≥5 mm
Tumor ≥10 mm
+Doxy (14d)
+ Doxy 10d
+ Doxy (+ ICI) 10d
- Doxy 14d
- Doxy (+ ICI) 14d
ICI
ICI
ICI (anti-CTLA4 plus anti-PD1)
Primary tumors
Weight
1
0.5
0
n=12
+ Doxy
+ Doxy + ICI
- Doxy
- Doxy + ICI
Metastasis
Weight
1
0.1
0.01
n=12
+ Doxy
+ Doxy + ICI
- Doxy
- Doxy + ICI
ROI 1=1.092e+09
+ Doxy
+ Doxy + ICI
- Doxy + ICI
- Doxy + ICI
- Doxy + ICI

Supplementary Figure 4

a. KRAS expression by PDAC-derived cell lines with doxycycline-inducible expression of KRASG12D (iKRAS). Cells were grown in the presence of dox (KRAS ON) or absence of dox (KRAS OFF) for 24 h prior to analysis. Tumors were induced with doxycycline for 20 days; then, iKras was turned OFF and tissues were harvested at the indicated time points. n = 2-3 mice/time point. Western blot image shows cells grown in tissue culture.

b. Box plots showing quantification of the change in tumor size in nude mice (n=9) and syngeneic FVB mice (n=20) maintained in the presence or absence of doxycycline for 14 days. Box plots show center line as median, box limits as upper and lower quartiles, and whiskers represent 1.5 × interquartile range (IQR).

c. Quantification of the change in CD3 T cells by IHC in tumors from FVB iKRAS mice (n=20) maintained in the presence or absence of doxycycline for 14 days.

d. Regression of peritoneal metastases in FVB iKRAS mice maintained in the absence of doxycycline (n=12). Box plots show center line as median, box limits as upper and lower quartiles, and whiskers represent 1.5 × interquartile range (IQR).

e. Immunohistochemistry of liver metastases in FVB iKRAS mice maintained in the presence or absence of doxycycline for 4 days.

f. Immune checkpoint inhibition (ICI) with anti-CTLA4 and anti-PD1 antibodies accelerated the regression of both primary tumors and metastases in mice maintained in the absence of doxycycline, but not in its presence. Box plots show center line as median, box limits as upper and lower quartiles, and whiskers represent 1.5 × interquartile range (IQR). The luciferase activity at experimental endpoint is shown.

a MΦ MARKERS

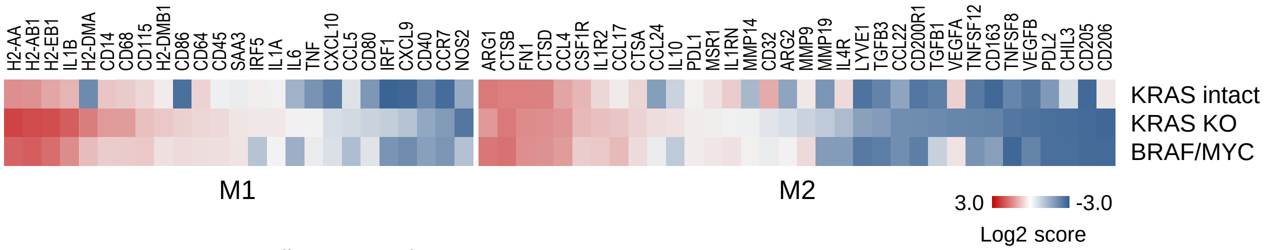

Median expression

Polarization	Intact	KO	BRAF/MYC
M1	0.85	1.44	1.1
M2	0.85	0.78	0.8

b T CELL MARKERS

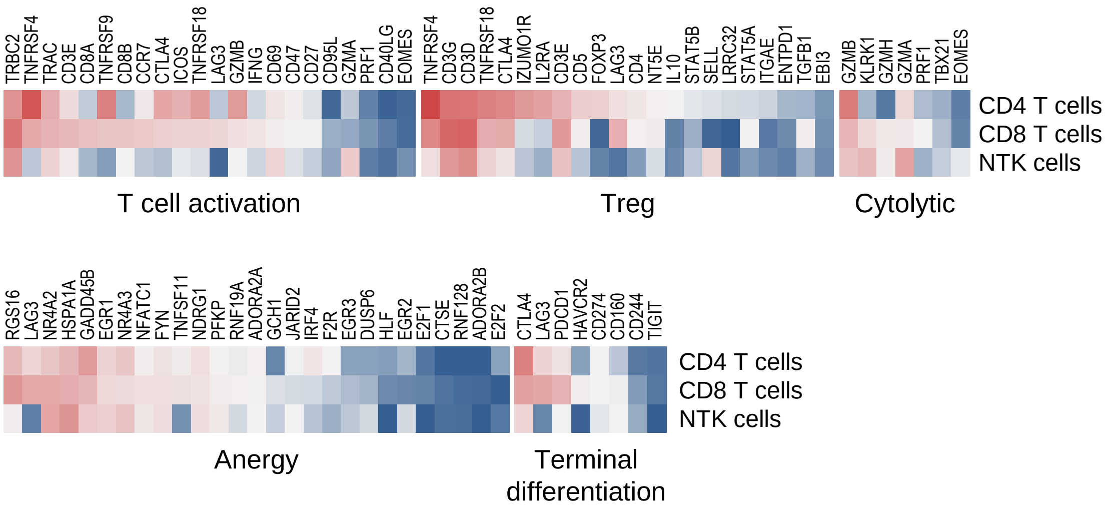

c T CELL MARKERS

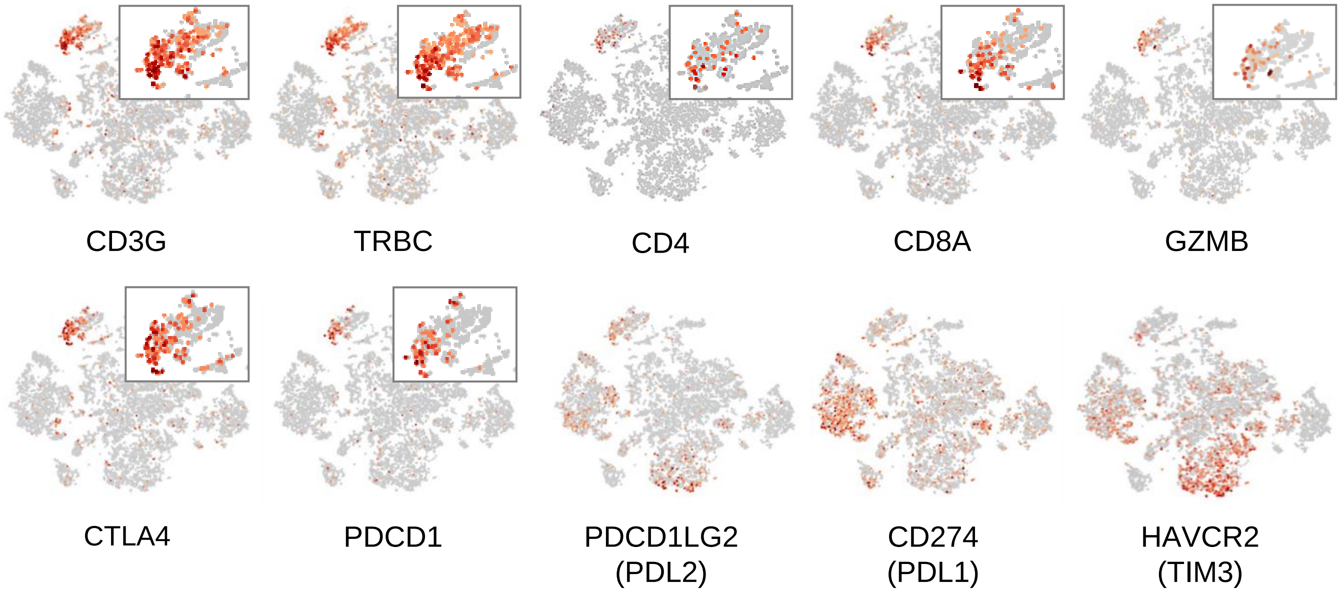

Supplementary Figure 5

d

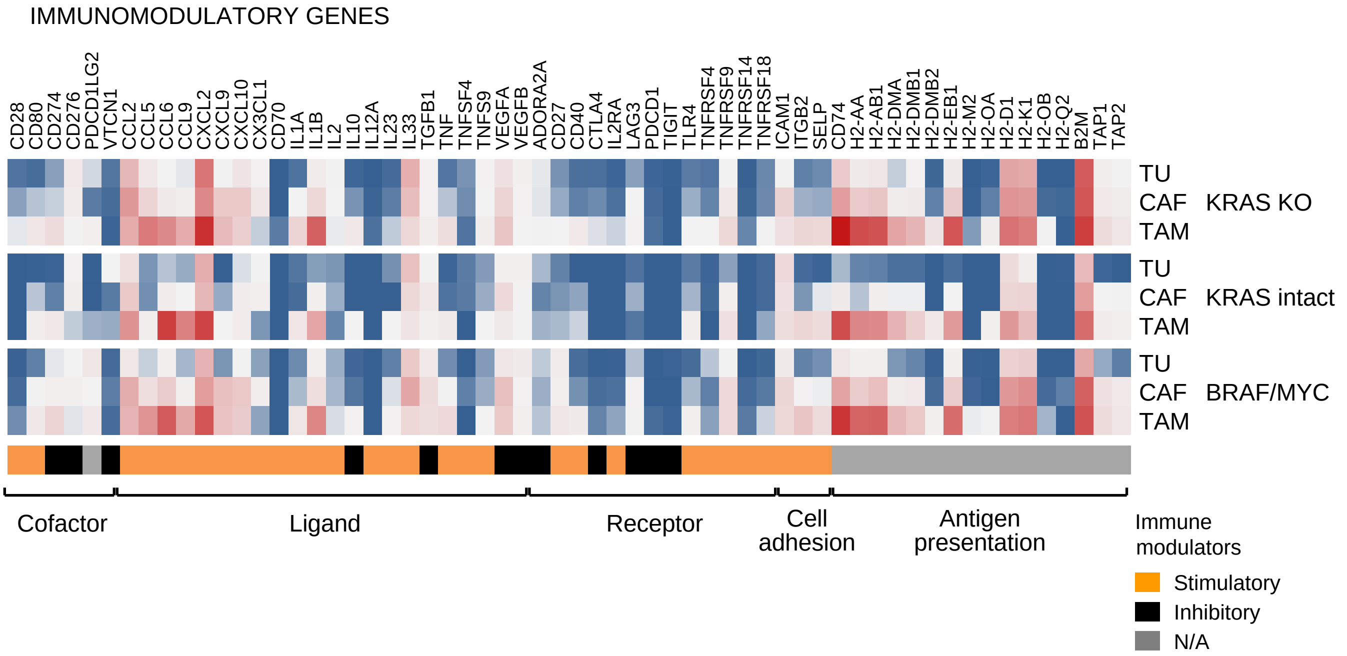

Supplementary Figure 5

a. Macrophage gene expression in KPC tumors. Median expression of M1 and M2 genes is shown.

b. Differential immune gene expression associated with T cell-enriched KRAS KO KPC tumors.

c. t-SNE plots showing expression of T cell specific genes in KRAS KO tumors.

d. Differential expression of immunomodulatory genes in KRAS intact and KRAS KO tumors.

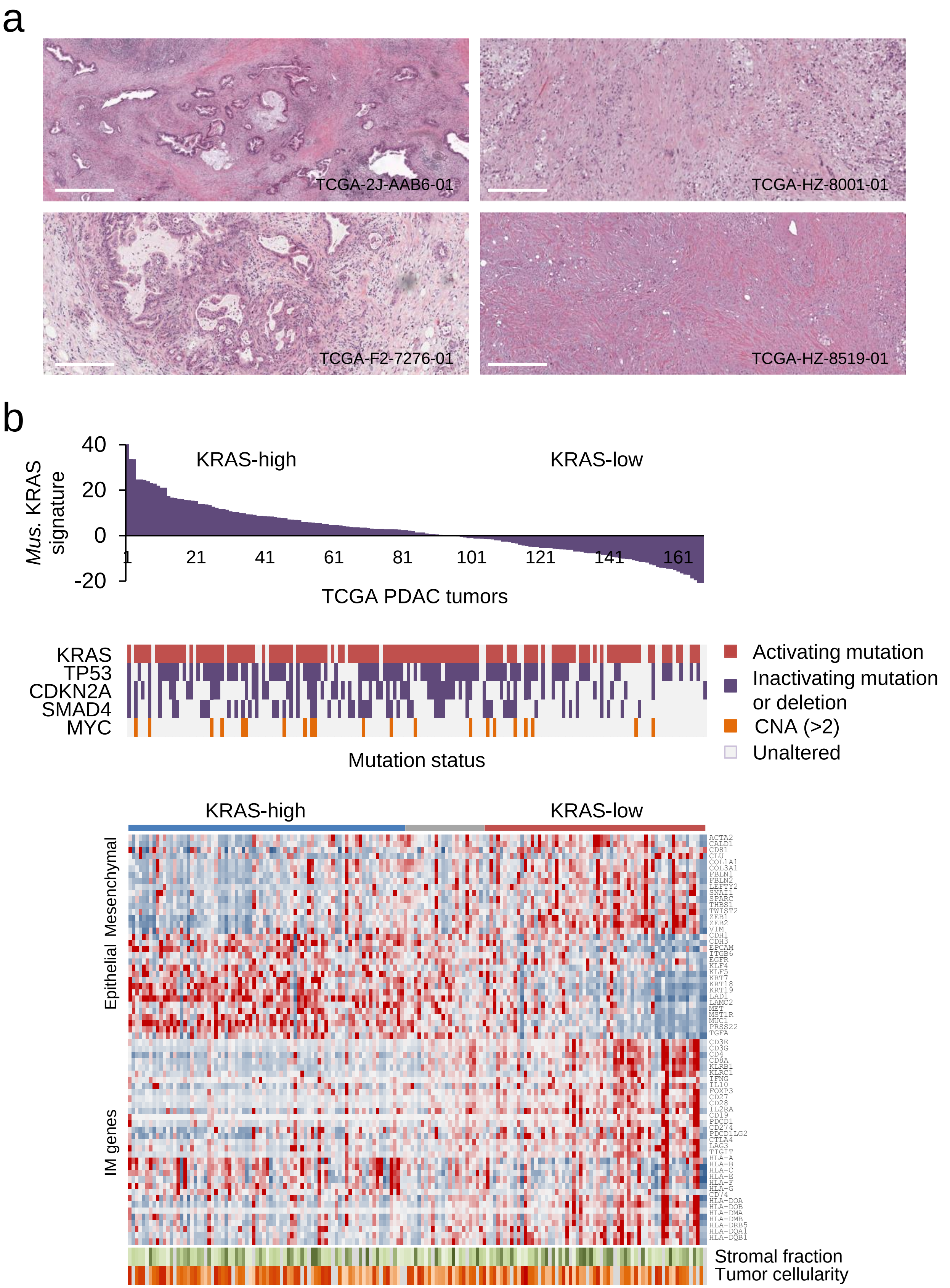

Supplementary Figure 6

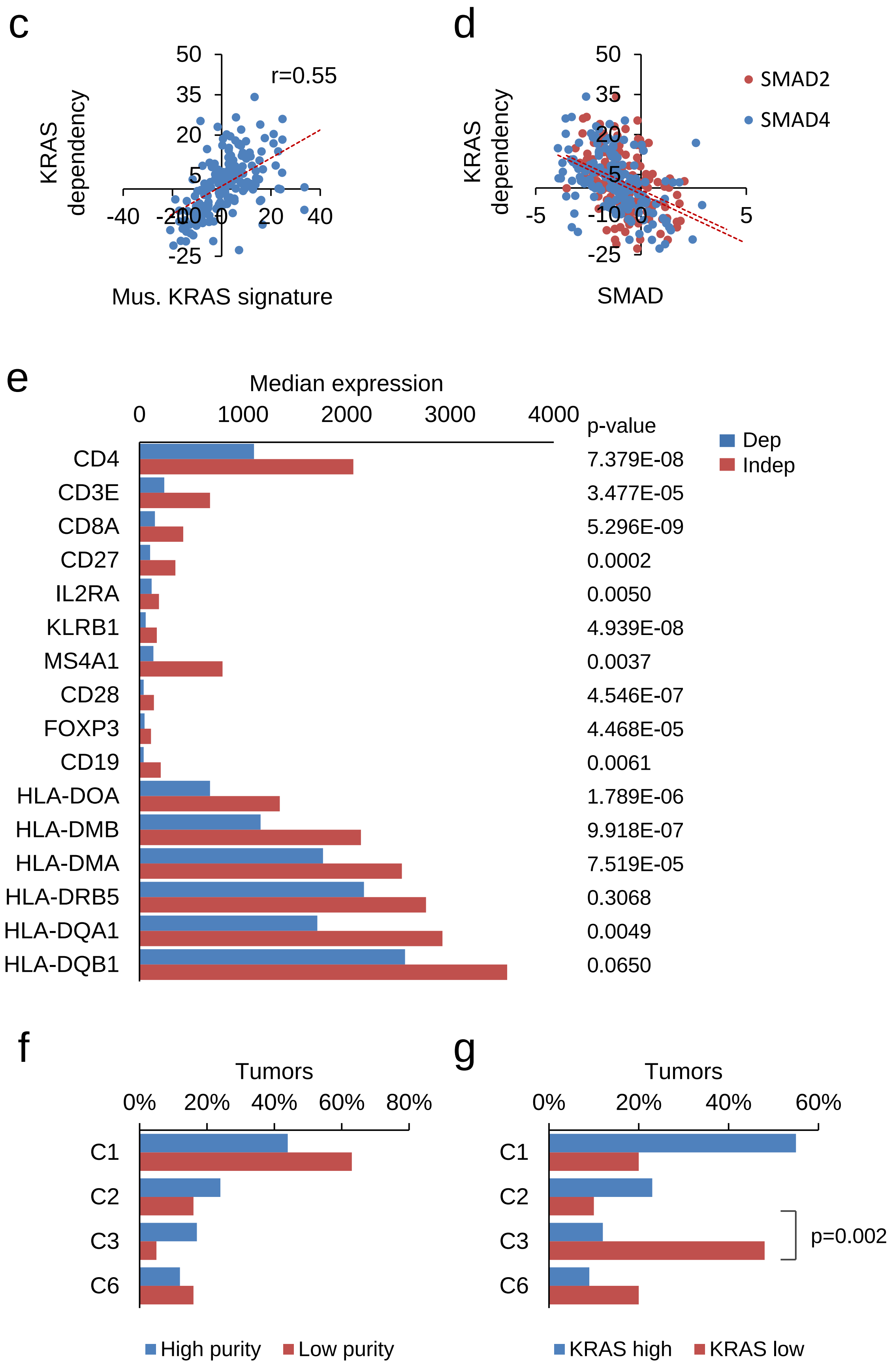

Supplementary Figure 6

h

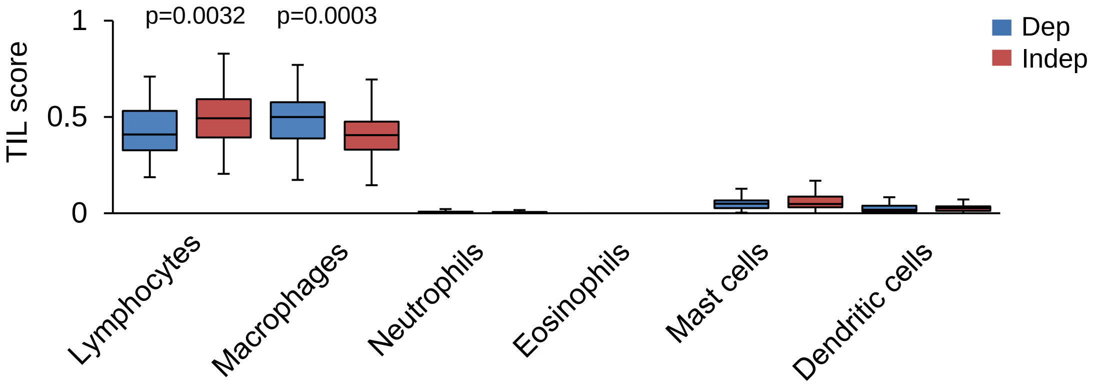
p=0.0032
p=0.0003
TIL score
1
0.5
0
Dep
Indep
Lymphocytes
Macrophages
Neutrophils
Eosinophils
Mast cells
Dendritic cells

i

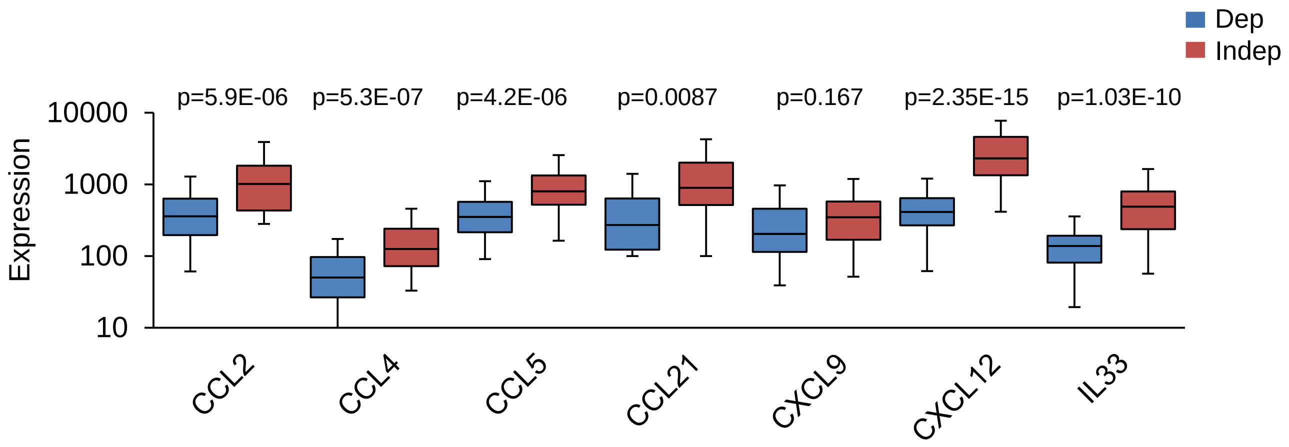
Dep
Indep
p=5.9E-06
p=5.3E-07
p=4.2E-06
p=0.0087
p=0.167
p=2.35E-15
p=1.03E-10
Expression
10000
1000
100
10
CCL2
CCL4
CCL5
CCL21
CXCL9
CXCL12
IL33

Supplementary Figure 6

a. Human PDAC samples were divided into two groups according to gene expression signatures that distinguish KRAS-dependent and KRAS-independent cell states. Representative tumors are shown. Scale bar 600 μM.

b. PDAC samples were divided into KRAS-high and KRAS-low groups based on the gene expression signature derived from genes differentially expressed in KRAS-intact tumors compared to cells from KO KPC tumors. Heatmaps depicting differentially expressed genes in KRAS-high versus KRAS-low tumors are shown.

c. Scatter plot comparing the two gene signature scores for human PDAC samples with Pearson correlation coefficient.

d. Scatter plot comparing SMAD2 and SMAD4 expression according to KRAS-dependency scores.

e. Differential expression of immunomodulatory genes in human PDAC samples (n=60 KRAS-dependent and 50 KRAS-independent tumors). Significance was determined using two-tailed test at the 0.05 confidence interval.

f. Distribution of immune subtypes within KRAS-dependent tumors with high cellularity (purity>0.38, n=41) and low cellularity (purity<0.38, n=19).

g. Distribution of immune subtypes within KRAS-high (n=60) and KRAS-low tumors (n=50): C1 (wound healing), C2 (IFNG dominant), C3 (inflammatory) and C6 (TGF-beta dominant). The proportion of samples belonging to each immune subtype is shown. Significance was determined using two-sided Fisher's exact test.

h. TIL subtypes associated with differential RAS dependency scores (n=60 KRAS-dependent and 50 KRAS-independent tumors). Box plots show center line as median, box limits as upper and lower quartiles, and whiskers represent 1.5 × interquartile range (IQR). Error bars represent the standard deviation. Significance was determined using two-tailed test at the 0.05 confidence interval.

i. Expression of cytokine genes associated with differential RAS dependency scores (n=60 KRAS-dependent and 50 KRAS-independent tumors). Box plots show center line as median, box limits as upper and lower quartiles, and whiskers represent 1.5 × interquartile range (IQR). Error bars represent the standard deviation. Significance was determined using two-tailed test at the 0.05 confidence interval.

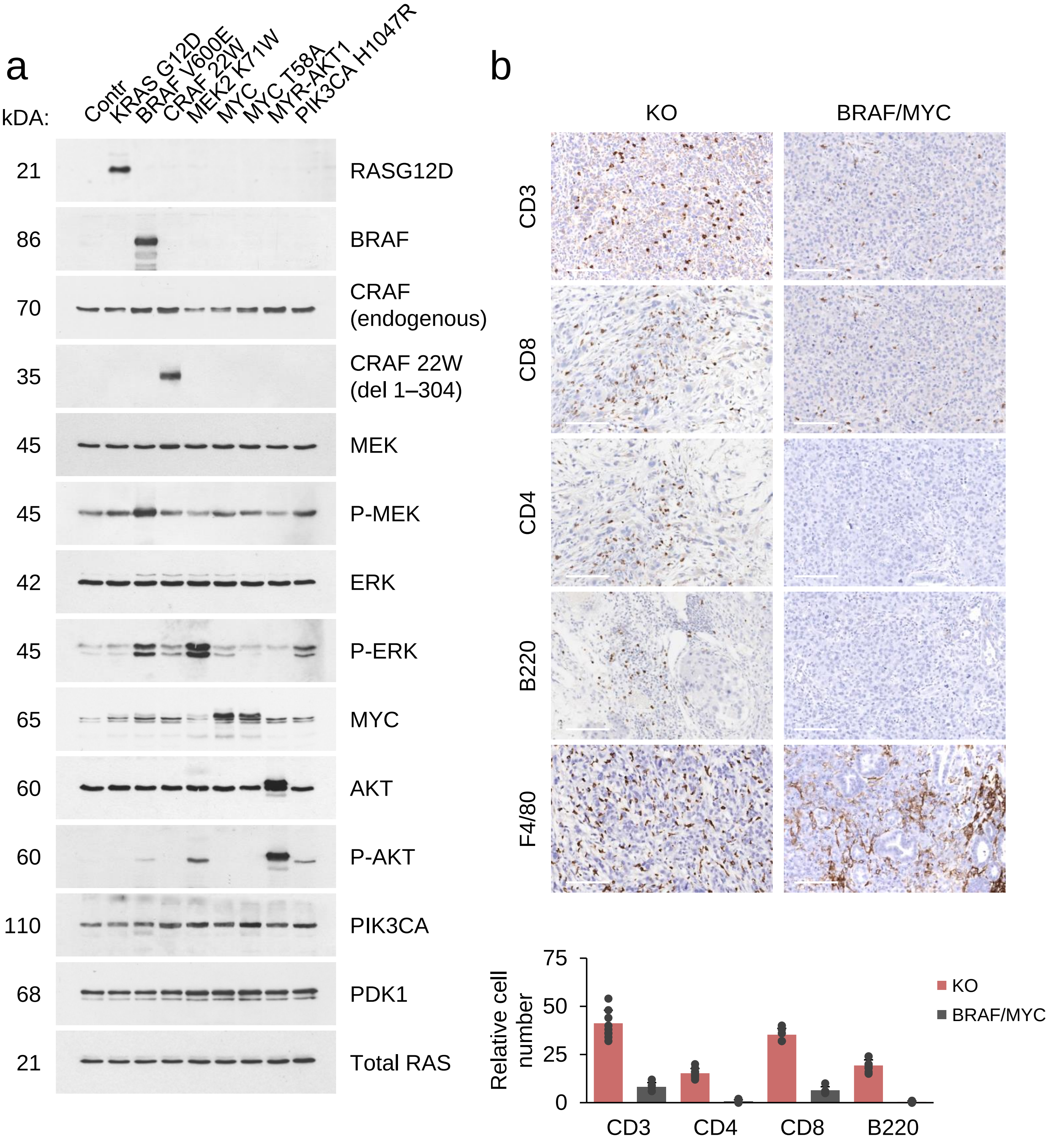

Supplementary Figure 7

c

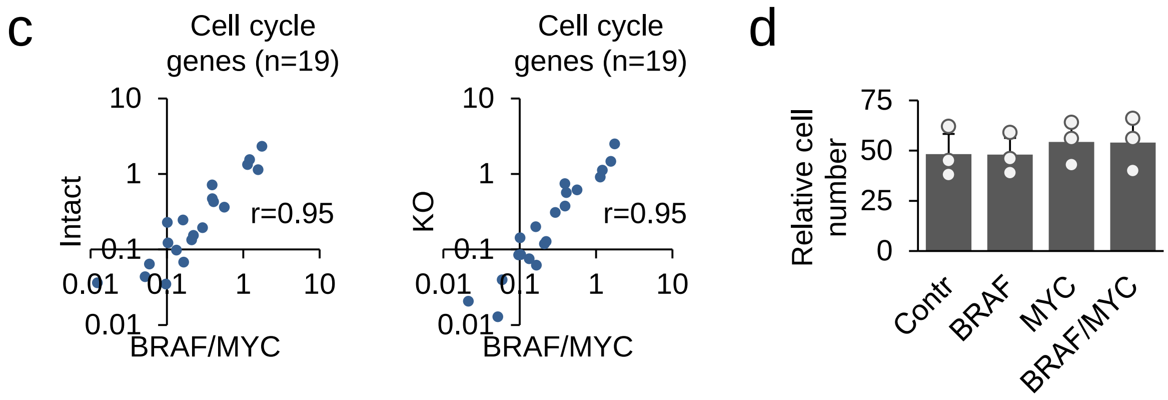

Cell cycle genes
CDKN1A, CDKN1B, CDKN2A, CDKN2B, CDKN2C, CCNE1, CCND1, CCND2, CCND3, CDK2, CDK4, CDK6, RB1, E2F1, E2F2, E2F3, PCNA, MKI67, MCM5

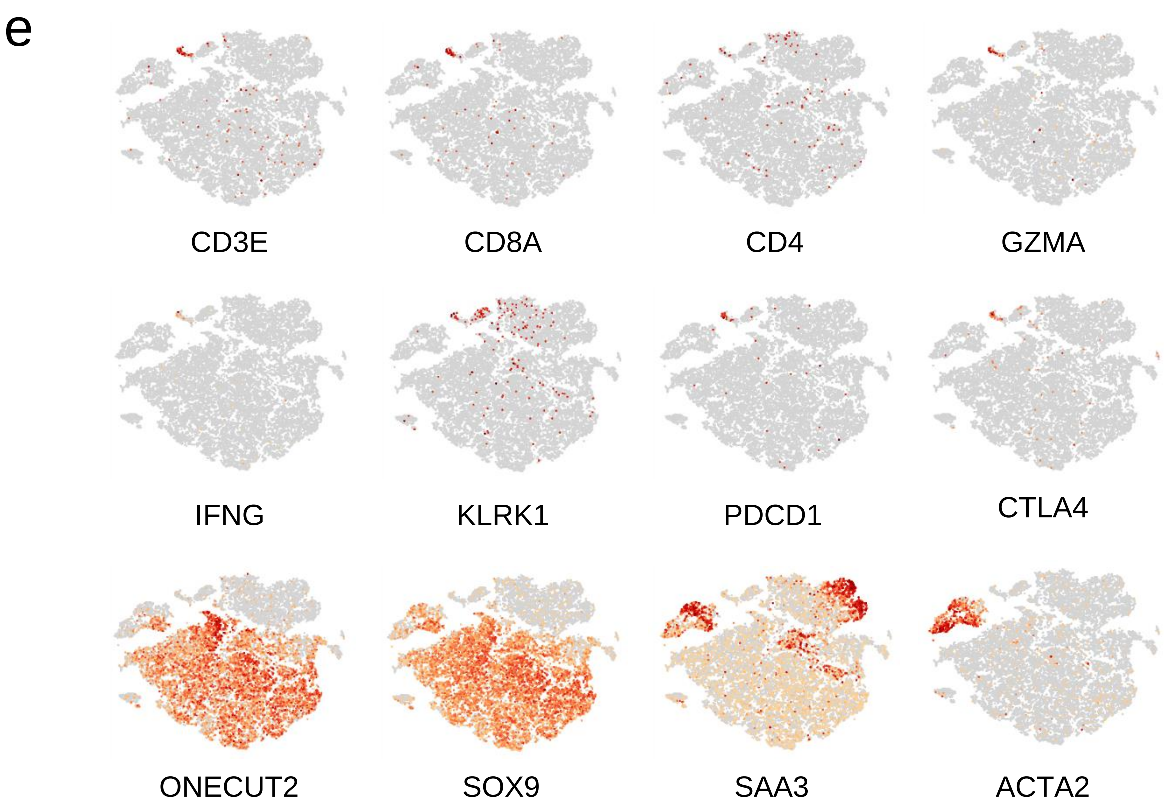

Supplementary Figure 7

a. Western blot analysis of key introduced components.

b. Immunohistochemistry of tumor-infiltrating lymphocytes in KRAS KO and BRAF/MYC reconstituted tumors (top). Scale bar 100 μM. Relative cell number per 10 high power fields is shown (bottom). Error bars represent the standard deviation.

c. Scatter plot comparing gene signature scores of cell cycle genes in KRAS intact, KRAS KO and BRAF/MYC transduced KRAS KO tumor cells with Pearson correlation coefficients.

d. Growth KRAS KO KPC cells transduced with the indicated genes was assessed in DME containing 5% FBS. Cumulative cell numbers after 2 weeks in culture are shown. Error bars represent the standard deviation.

e. t-SNE plots showing expression of T cell specific genes in BRAF/MYC KRAS KO tumors.

Supplementary References

1. Ischenko, I., Petrenko, O. & Hayman, M. J. Analysis of the tumor-initiating and metastatic capacity of PDX1-positive cells from the adult pancreas. Proceedings of the National Academy of Sciences of the United States of America 111, 3466-3471, doi:10.1073/pnas.1319911111 (2014).

2. Hingorani, S. R. et al. Trp53R172H and KrasG12D cooperate to promote chromosomal instability and widely metastatic pancreatic ductal adenocarcinoma in mice. Cancer cell 7, 469-483, doi:10.1016/j.ccr.2005.04.023 (2005).

3. Sivaram, N. et al. Tumor-intrinsic PIK3CA represses tumor immunogenecity in a model of pancreatic cancer. The Journal of clinical investigation 129, 3264-3276, doi:10.1172/JCI123540 (2019).

4. Collins, M. A. et al. Oncogenic Kras is required for both the initiation and maintenance of pancreatic cancer in mice. The Journal of clinical investigation 122, 639-653, doi:10.1172/JCI59227 (2012).
