# Supplementary material for: KRAS drives immune evasion in a genetic model of pancreatic cancer
Source: Nat Commun. 2021 Mar 5;12:1482. doi: 10.1038/s41467-021-21736-w (PMC7935870; doi:10.1038/s41467-021-21736-w)
Supplement: Supplementary file 2 — Reporting Summary [file 41467_2021_21736_MOESM2_ESM.pdf]

nature research

Corresponding author(s): Oleksi Petrenko, Nancy Reich

Last updated by author(s): Nov 15, 2020

Reporting Summary

Nature Research wishes to improve the reproducibility of the work that we publish. This form provides structure for consistency and transparency in reporting. For further information on Nature Research policies, see our Editorial Policies and the Editorial Policy Checklist.

Statistics

For all statistical analyses, confirm that the following items are present in the figure legend, table legend, main text, or Methods section.

n/a	Confirmed	
☐	☒	The exact sample size (n) for each experimental group/condition, given as a discrete number and unit of measurement
☐	☒	A statement on whether measurements were taken from distinct samples or whether the same sample was measured repeatedly
☐	☒	The statistical test(s) used AND whether they are one- or two-sided<br>*Only common tests should be described solely by name; describe more complex techniques in the Methods section.*
☒	☐	A description of all covariates tested
☒	☐	A description of any assumptions or corrections, such as tests of normality and adjustment for multiple comparisons
☐	☒	A full description of the statistical parameters including central tendency (e.g. means) or other basic estimates (e.g. regression coefficient) AND variation (e.g. standard deviation) or associated estimates of uncertainty (e.g. confidence intervals)
☐	☒	For null hypothesis testing, the test statistic (e.g. F, t, r) with confidence intervals, effect sizes, degrees of freedom and P value noted<br>*Give P values as exact values whenever suitable.*
☒	☐	For Bayesian analysis, information on the choice of priors and Markov chain Monte Carlo settings
☒	☐	For hierarchical and complex designs, identification of the appropriate level for tests and full reporting of outcomes
☐	☒	Estimates of effect sizes (e.g. Cohen's d, Pearson's r), indicating how they were calculated

Our web collection on statistics for biologists contains articles on many of the points above.

Software and code

Policy information about availability of computer code

Data collection	10 x Genomics Cell Ranger pipeline (version 3.0.1), Loupe Cell Browser v3.0.1, BD CellQuest (Becton Dickinson)
Data analysis	10 x Genomics Loupe Cell Browser (version 3.0.1), Loupe Cell Browser v3.0.1, Image Studio v3.1 (LI-COR), R Project for Statistical Computing (The R foundation), ELDA (www.elda.at), BD CellQuest (Becton Dickinson)

For manuscripts utilizing custom algorithms or software that are central to the research but not yet described in published literature, software must be made available to editors and reviewers. We strongly encourage code deposition in a community repository (e.g. GitHub). See the Nature Research guidelines for submitting code & software for further information.

Data

Policy information about availability of data

All manuscripts must include a data availability statement. This statement should provide the following information, where applicable:

- Accession codes, unique identifiers, or web links for publicly available datasets
- A list of figures that have associated raw data
- A description of any restrictions on data availability

Pancreatic adenocarcinoma clinical data and expression profiles (TCGA, provisional) were downloaded from cBioPortal (http://www.cbioportal.org). The RNA-Seq data generated in this study have been deposited in the GEO/SRA database under accession code GSE132582 [https://www.ncbi.nlm.nih.gov/geo/query/acc.cgi?acc=GSE132582]. The single cell sequenicjng data are deposited in the GEO/SRA database under accession code GSE146694 [https://www.ncbi.nlm.nih.gov/geo/query/acc.cgi?acc=GSE146694]. Source data are available as a Source Data file. The remaining data are available within the Article, Supplementary Information or available from the authors upon request.

Field-specific reporting

Please select the one below that is the best fit for your research. If you are not sure, read the appropriate sections before making your selection.

☒ Life sciences ☐ Behavioural & social sciences ☐ Ecological, evolutionary & environmental sciences

For a reference copy of the document with all sections, see nature.com/documents/nr-reporting-summary-flat.pdf

Life sciences study design

All studies must disclose on these points even when the disclosure is negative.

Sample size	Sample size used in the study was determined based on the need to provide sufficient statistical power.
Data exclusions	No data were excluded from the analysis.
Replication	Multiple replicates and repetitions were performed. A minimum of three independent sample replicates were tested for Western blotting and histology, and multiple mice were tested as indicated in figures. All attempts at replication were successful.
Randomization	We used both male and female mice in the study. There were no physical attributes that influenced whether the animal was used for control or test samples. Random cell clones with altered genotypes were evaluated. No external or internal factors changed during the period of experimentation.
Blinding	The samples were identified prior to testing, however the results of tumor formation and the analyses of protein expression and immunohistochemistry were evaluated independently by at least two persons. The results of scRNASeq were evaluated independently by at least two persons.

Reporting for specific materials, systems and methods

We require information from authors about some types of materials, experimental systems and methods used in many studies. Here, indicate whether each material, system or method listed is relevant to your study. If you are not sure if a list item applies to your research, read the appropriate section before selecting a response.

Materials & experimental systems

n/a	Involved in the study	
☐	☒	Antibodies
☐	☒	Eukaryotic cell lines
☒	☐	Palaeontology and archaeology
☐	☒	Animals and other organisms
☒	☐	Human research participants
☒	☐	Clinical data
☒	☐	Dual use research of concern

Methods

n/a	Involved in the study	
☒	☐	ChIP-seq
☐	☒	Flow cytometry
☒	☐	MRI-based neuroimaging

Antibodies

Antibodies used

Ras (G12D Mutant Specific) (D8H7) Rabbit mAb (dilution 1:2000) Cell Signaling Technology Cat#14429;
Rabbit polyclonal anti-AKT (dilution 1:2000) Cell Signaling Technology Cat#9272;
Phospho-Akt (Thr308) (244F9) Rabbit mAb (dilution 1:500) Cell Signaling Technology Cat#4056;
Phospho-Akt (Ser473) (D9E) XP® Rabbit mAb (dilution 1:500) Cell Signaling Technology Cat#4060;
B-Raf (55C6) Rabbit mAb (dilution 1:500) Cell Signaling Technology Cat#9433;
c-Raf Rabbit Antibody (dilution 1:500) Cell Signaling Technology Cat#9422;
Phospho-MEK1/2 (Ser217/221) Rabbit Antibody (dilution 1:1000) Cell Signaling Technology Cat#9121;
p44/42 MAPK (Erk1/2) Rabbit Antibody (dilution 1:1000) Cell Signaling Technology Cat#9102;
Phospho-p44/42 MAPK (Erk1/2) (Thr202/Tyr204) (D13.14.4E) XP® Rabbit mAb (dilution 1:1000) Cell Signaling Technology Cat#4370;
PI3 Kinase p110α (C73F8) Rabbit mAb (dilution 1:1000) Cell Signaling Technology Cat#4249;
Phospho-GSK-3α/β (Ser21/9) Antibody (dilution 1:500) Cell Signaling Technology Cat#9331;
Keratin 17/19 (D32D9) Rabbit mAb #3984 (dilution 1:1000) Cell Signaling Technology Cat#3984;
Sox9 (D8G8H) Rabbit mAb #82630 (dilution 1:1000) Cell Signaling Technology Cat#82630;
K-Ras (F234) mAb (dilution 1:200) Santa Cruz Biotechnology Cat#sc-30;
c-Myc Rabbbit Antibody (N-262) (dilution 1:1000) Santa Cruz Biotechnology Cat#sc-764;
Anti-phospho-c-Myc (Thr58/Ser62) Rabbit mAb (dilution 1:2000) Millipore Sigma Cat#04-217;
Anti-MAP Kinase 2/Erk2 Antibody clone 1B3B9 mAb (dilution 1:2000) EMD Millipore Cat#05-157;
Anti-PDX-1 mouse mAb (dilution 1:2000) BD Biosciences Cat#562160;

	Anti-ERK1/2 Phospho (Thr202/Tyr204) Antibody (dilution 1:200) BioLegend Cat#675502;<br>CD326 (EpCAM) Monoclonal Antibody (1B7), PE (dilution 1:1000) eBioscience Cat#12-9326-42;<br>Ly-6A/E (Sca-1) Monoclonal Antibody (D7), APC (dilution 1:1000) eBioscience Cat#17-5981-81;<br>Anti-PD1 (dilution 1:10) BioXCell Cat#BE0146;<br>Anti-CTLA4 (dilution 1:10) BioXCell Cat#BE0164
Validation	Antibodies were purchased from reputable commercial companies that stated validation of their products. We tested antibodies periodically by Western blotting with control cells in comparison with cells lacking expression of the specific protein. We also compared the antibody results with alternative antibodies. Immunohistochemistry was performed by a commercial service (HistoWiz) that included positive and negative controls for antibodies.

Eukaryotic cell lines

Policy information about cell lines

Cell line source(s)	We used KRASG12D p53KO cell lines (KP) from Ischenko et al.,PNAS. 2014; KRASG12D p53R172H cell lines (KPC) from Dr. David Tuveson, Cold Spring Harbor Laboratory); iKRAS p53R172H/+ cell lines (A9312) from Dr. Marina Pasca di Magliano, University of Michigan; Phoenix E cells from Dr. Ihor Lemischka, Princeton University, and 293T cell line from ATCC.
Authentication	Cell lines that we generated were authenticated by DNA sequencing of intentionally altered genes. Maintenance of genotype was periodically tested by immunohistochemistry and Western blotting. Multiple subcloning was performed to ensure homogeneity of the cell populations. RNA expression of genes that we have modified was tested periodically.
Mycoplasma contamination	Cell lines tested negative for mycoplasma contamination
Commonly misidentified lines<br>(See ICLAC register)	No commonly misidentified cell lines were used in the study

Animals and other organisms

Policy information about studies involving animals; ARRIVE guidelines recommended for reporting animal research

Laboratory animals	Animals were purchased from Jackson laboratory. We used NU/J, C57BL/6J, FVB/NJ, CD4 KO and CD8 KO mice between 6-10 weeks of age. Housing of animals was maintained in the Division of Animal Laboratory Research at Stony Brook University facility. Temperature, humidity, dark/light cycles were controlled as optimum for animal well-being. Housing conditions included a 12 hour light/12 hour dark cycle with temperatures of 65-75°F (~18-23°C) and 40-60% humidity. Mice were housed together when possible.
Wild animals	No wild animals were used in the study
Field-collected samples	No field collected samples were used in the study
Ethics oversight	The Division of Animal Laboratory Research at Stony Brook University operates in accordance with the American Association for Laboratory Animal Science (AALAS), the American College of Laboratory Animal Medicine (ACLAM), and Animal Welfare Assurance ID D16-00006 (A3011-01) of the National Institutes of Health (NIH). This study was carried out in strict accordance with the recommendations in the Guide for the Care and Use of Laboratory Animals of the NIH. The protocol was approved by the Institutional Animal Care and Use Committee of Stony Brook University (IACUC #2011-0356).

Note that full information on the approval of the study protocol must also be provided in the manuscript.

Flow Cytometry

Plots

Confirm that:

- [x] The axis labels state the marker and fluorochrome used (e.g. CD4-FITC).
- [x] The axis scales are clearly visible. Include numbers along axes only for bottom left plot of group (a 'group' is an analysis of identical markers).
- [x] All plots are contour plots with outliers or pseudocolor plots.
- [x] A numerical value for number of cells or percentage (with statistics) is provided.

Methodology

Sample preparation	Cell were extracted from mouse tumor tissues using Collagenase Hyaluronidase (Stemcell). Cells were suspended at a density of 10exp6 cells/ml in PBS. Direct staining of cells was performed with the vendor-recommended dilution of fluorescent labeled antibody for 30 minutes at 4C. Cells were centrifuged and washed in PBS and data was acquired within 1 hour after preparation.
Instrument	FACSCalibur (BD)

Software	BD CellQuest (Becton Dickinson)
Cell population abundance	There was no cell sorting involved
Gating strategy	Forward/side scatter for live cells

☒ Tick this box to confirm that a figure exemplifying the gating strategy is provided in the Supplementary Information.
